# Exploratory study to evaluate the acceptability of a wearable accelerometer to assess motor progression in motor neuron disease

**DOI:** 10.1007/s00415-024-12449-3

**Published:** 2024-05-28

**Authors:** Emily Beswick, Alexander Christides, Alexander Symonds, Micheaela Johnson, Thomas Fawcett, Judith Newton, Dawn Lyle, Christine Weaver, Siddharthan Chandran, Suvankar Pal

**Affiliations:** 1 Centre for Clinical Brain Sciences, The University of Edinburgh, Edinburgh, Scotland; 2 Anne Rowling Regenerative Neurology Clinic, The University of Edinburgh, 49 Little France Crescent, Edinburgh, EH16 4SB UK; 3 Euan MacDonald Centre for MND Research, The University of Edinburgh, Edinburgh, Scotland; 4 The School of Medicine and Veterinary Medicine, The University of Edinburgh, Edinburgh, Scotland; 5 UK Dementia Research Institute, The University of Edinburgh, Edinburgh, Scotland

**Keywords:** Wearable devices, Accelerometer, Motor neuron disease, Digital health

## Abstract

Motor neuron disease (MND) is a rapidly progressive condition traditionally assessed using a questionnaire to evaluate physical function, the revised amyotrophic lateral sclerosis functional rating scale (ALSFRS-R). Its use can be associated with poor sensitivity in detecting subtle changes over time and there is an urgent need for more sensitive and specific outcome measures. The ActiGraph GT9X is a wearable device containing multiple sensors that can be used to provide metrics that represent physical activity. The primary aim of this study was to investigate the initial suitability and acceptability of limb-worn wearable devices to group of people with MND in Scotland. A secondary aim was to explore the preliminary associations between the accelerometer sensor data within the ActiGraph GT9X and established measures of physical function. 10 participants with MND completed a 12-week schedule of assessments including fortnightly study visits, both in-person and over videoconferencing software. Participants wore the device on their right wrist and right ankle for a series of movements, during a 6-min walking test and for a period of 24-h wear, including overnight. Participants also completed an ALSFRS-R and questionnaires on their experience with the devices. 80% of the participants found wearing these devices to be a positive experience and no one reported interference with daily living or added burden. However, 30% of the participants experienced technical issues with their devices. Data from the wearable devices correlated with established measures of physical function.

## Introduction

Motor neuron disease (MND) is a rapidly progressive, fatal neurodegenerative condition, currently without a cure [1]. The clinical features of MND include muscle weakness, wasting and spasticity, impacting on mobility and activities of daily living. This ultimately results in speech and swallowing difficulties, and death from respiratory insufficiency. MND is often referred to by the most common sub-type of the disease, amyotrophic lateral sclerosis, but MND encompasses several disease sub-types [2].

Disease progression is traditionally assessed in clinical practice and trials using the revised amyotrophic lateral sclerosis functional rating scale (ALSFRS-R) administered at face-to-face appointments [3]. Lower scores indicate more severe dysfunction, and the scale is widely used as a primary outcome, or co-primary outcome alongside survival, in clinical trials [4]. Whilst the ALSFRS-R remains the internationally recognised benchmark of disease progression in MND, concerns have been raised over its sensitivity in earlier [5] and later [3] stages of the disease, with improvements on the scale evident when symptomatic therapy is introduced [6, 7] despite the disease continuing to progress. These issues of the scale as an outcome measure in turn necessitate large numbers of participants and longer duration trials. There is an urgent need for more sensitive and specific outcome measures, suitable for remote monitoring, to objectively measure physical functioning and disease progression in MND for trial participants.

Remote monitoring of motor functioning offers an opportunity for trial participants with MND to gather information relating to disease progression in the community between scheduled study visits. The information obtained from wearable devices may also enable trialists to require fewer in-person assessments, reducing the travel burden that trial participation often places on people with MND and their caregivers.

Wearable devices have the potential to quantify activity and map a range of motor symptoms, including gait disturbance and impairment in upper limb strength, movement, and dexterity. This may be particularly beneficial in diseases such as MND where presentation and progression are heterogeneous. These devices have been successfully used to quantify mobility and activities of daily living in people with Parkinson’s disease [8], multiple sclerosis [9], and stroke [10] and offer promise for MND assessment [11].

Accelerometers are a type of sensor in wearable devices that are particularly suited to assessment of physical function, monitor decline and recording time spent active [12]. The data obtained from accelerometers can be correlated with the level of change expected on standardised tests of disease progression and functionality, primarily the ALSFRS-R in the context of MND research [13].

The ActiGraph GT9X device is an inertial measurement unit that comprises various sensors, specifically accelerometers, gyroscopes and magnometers. The ActiGraph GT9X can be worn on the chest, limbs or waist to evaluate movement and activity in free-living conditions, with optimal wear location dependent on which functional outcome is being evaluated [14]. The device’s accelerometery sensor collects raw accelerometery ‘counts’ in three axes (A1, A2 and A3) and a combination of all three axes (vector magnitude (VM)). The devices also collect data on step count, metabolic equivalents (METs) and percentage of time in moderate-to-vigorous physical activity (MVPA).

The ActiGraph GT9X was selected for the current study over other wearables devices containing accelerometery sensors due to previous evidence of use of this device in people with MND [11, 14, 15] and initial promising feedback of low burden for this participant group [12].

Previous research has highlighted strong associations between daily-wear endpoints of the accelerometer in the ActiGraph GT9X (average daytime active, percentage of daytime active, total daytime activity score and total 24-h activity score) and ALSFRS-R scores for up to 21 months [12]. Data from the ActiGraph GT9X devices worn during motor tasks have also offered initial promise in predictive value of overall ALSFRS-R decline [15] and limb-specific sub-scores [16]. Real-time wear data may also provide clinically relevant, region-specific, measures of function that are more sensitive than the ALSFRS-R at detecting change and more ecologically valid [11].

However, the primary focus of previous research using the ActiGraph GT9X, and other wearable devices containing accelerometer sensors, in cohorts with MND has been the viability of these devices to evaluate function, with limited focus on the acceptability to participants. Exploring the acceptability of these devices in different wear locations and overnight, for use during remote and in-person assessment, adds a person-centred perspective to future consensus on the suitability of these devices to people with MND.

### Aim

This was a single-centre, exploratory pilot study at the Anne Rowling Regenerative Neurology Clinic, University of Edinburgh, with the primary aim of investigating the suitability and acceptability of limb-worn wearable devices to group of Scottish people with MND. A secondary aim was to explore the preliminary associations between accelerometer sensor data from these devices and established measures of physical function.

## Methods

### Participants

Ten people with a confirmed diagnosis of MND by El Escorial criteria were invited to participate. All had provided prior consent to be contacted about ongoing research projects on the Scottish CARE-MND (Clinical Audit Research and Evaluation) register [17] and met study inclusion criteria as outlined in Table 1. All provided informed written consent ahead of participating.

**Table 1:** Study inclusion and exclusion criteria

Inclusion	Exclusion
Ambulant at the time of recruitment Participant can be using NIV, gastrostomy and communicative devices Participant must have arm and leg function sufficient to complete required motor assessments	People with slowly progressing sub-types of MND including PLS and PMA, and long surviving ALS (> 7 years post diagnosis) will be accepted at the investigator’s discretion Conditions other than MND which may impact on upper and lower limb functioning Significant cognitive impairment Receiving invasive ventilation Implantable cardiac device in situ

### Study schedule and assessments

Participants completed a 12-week schedule of assessments with fortnightly study visits, shown in Table 2. The weeks 0, 6 and 12 visits were in-person, with the remainder of study visits completed remotely using videoconferencing software. At the weeks 0, 6 and 12 visits participants completed questionnaires (Appendix 1) to provide feedback on their experience of the wearables devices and participating in the study. A researcher-led ALSFRS-R (Appendix 2) was conducted at the beginning and end of the study period.

**Table 2:** Study assessment schedule

Week	Location	Assessments
0	In-person	Screening for eligibility Informed consent process and opportunity to ask questions Information on using the devices and appointment schedule Questionnaire 1: expectations and concerns on device use First set of motor assessments Complete ALSFRS-R with researcher Day after the appointment—wear the device for 24 h (from the time they wake up, all day and overnight until they wake the next day)
2	Video conferencing	Fortnightly motor assessment series Day after the appointment—wear the device for 24 h
4	Video conferencing	Fortnightly motor assessment series Day after the appointment—wear the device for 24 h
6	In-person	Questionnaire 2 on the participant’s experience so far Fortnightly motor assessment Research team to check data recording, charge and download data Day after the appointment—wear the device for 24 h
8	Video conferencing	Fortnightly motor assessment series Day after the appointment—wear the device for 24 h
10	Video conferencing	Fortnightly motor assessment series Day after the appointment—wear the device for 24 h
12	In-person	Day before the appointment—wear the device for 24 h Final motor assessment Complete ALSFRS-R with researcher Participant returns devices to the research team Questionnaire 3 on their experience of wearing devices

At each study visit, participants completed a standardised series of movements whilst wearing one ActiGraph GT9X on their right wrist, and one on their right ankle. These movements included raising both arms above their head, closing and opening their right fist and alternate supination and pronation of the right hand.

Participants were then asked to complete a 6-min walking test (6MWT), a standardised assessment paradigm validated as an alternative outcome measure in MND [18], with the distance walked measured during in-person visits. This test involves asking the participant to walk and measuring the distance that they are able to cover during the 6-min timeframe. To explore the suitability of these devices for use during a walking task in the remote study assessments, a 6MWT was completed both during in-person and remote visits. During the in-person study visits, a trundle wheel was used to measure the distance walked during the 6MWT. At the remote visits, the primary focus was on the acceptability of the devices to participants for wearing during a walking task and distance walked was not measured.

The day after each of the in-person or remote appointments where motor assessments were completed (or the day before the final appointment) participants were asked to wear both of their ActiGraph devices for 24 h. This was defined as from when they woke that morning, throughout the day, overnight and taking them off when they woke the next morning. The lower limb device was secured on the participant’s right ankle and the upper limb device on their right wrist.

Additional clinical and demographic data were requested from each individual’s CARE-MND record, available in Appendix 3.

### Wearable device selection

The wearable device used in this study was the ActiGraph GT9X. This device was selected as there have been several previous studies that supported the potential utility of ActiGraph accelerometers in people with MND [12, 15]. These studies have shown strong associations between daily-wear accelerometer endpoints (average daytime active, percentage of daytime active, total daytime activity score and total 24-h activity score) and ALSFRS(R) scores for up to 21 months when evaluated using the ActiGraph. Further, ActiGraph accelerometer data were strongly associated with the ALSFRS-R data, but indicated less variability over time [12].

The ActiGraph was chosen over other devices as previous research suggested that this device may also be able to monitor disease progression, providing preliminary evidence of an ability to evaluate motor symptom changes over time [12]. ActiGraph data from limb-specific motor exercises in a large cohort of people with MND offered predictive value of ALSFRS(R) decline when used in machine learning models [15]. Ultimately, the decision to use this device was influenced by initially promising findings on the acceptability of wearing these devices to people with MND, with a previous study reporting wear-time adherence of 93% and a mean rating of burden at 1.3, on a scale of 0 (low burden) to 10 (high burden) [12].

In the current study, only the accelerometer sensor was enabled out of the full inertial measurement unit’s sensors due to battery life concerns. The sensor was used to provide information on acceleration, general activity, and step count. Each participant received two ActiGraph GT9X devices for the duration of their involvement in the study. These devices were worn by the participants on their right wrist and right ankle, affixed by a rubber watch strap or a Velcro strap.

### Questionnaires investigating acceptability

Novel questionnaires investigating the experience and acceptance of devices were designed specifically for this study and these are available in Appendix 1. These questionnaires contained a series of statements regarding potential benefits, concerns and barriers to using wearable devices, with ‘Yes’, ‘No’ and ‘Unsure’ options for participants to indicate their level of agreement. Each questionnaire also included a free-text response item for participants to provide any additional comments or feedback if they wished to do so. Feedback on the suitability of these questions was sought from people with MND, their caregivers and clinical team prior to use.

### Analysis plan

Data from the devices were analysed by in ActiLife software provided by ActiGraph, using ActiLife’s established analysis algorithms. When the accelerometer sensor is enabled, the ActiGraph devices collect raw accelerometery ‘counts’ in three axes (A1, A2 and A3) and these axes are combined and represented as vector magnitude (VM) count, with a higher VM count indicative of greater movement.

To provide an overall measure of daily activity, the total VM counts from ankle and wrist-worn devices were combined from 24-h wear periods. To evaluate walking capacity, the VM counts from only ankle-worn devices were explored during the time periods where motor assessments were undertaken.

Statistical analyses were completed using R Studio. Wear time was calculated as the percentage of time within the 24-h period that both of the devices were worn and compared between different device wear locations using the Wilcoxon rank-sum test. Wilcoxon rank-sum test was also used to explore differences between remote and in-person 6MWT. Linear regression was used to explore associations between device data and ALSFRS-R scores, and between ankle VM counts and distance walked in the 6MWT.

## Results

### Characteristics of participants

Ten individuals with MND participated in this study. Average age at participation was 62 (SD = 12). Eight participants were male, and two were categorised as long survivors (diagnosed over 8 years ago [19]) with bulbar onset in one individual. All participants completed the full 12 weeks of data collection with no missing study visits or 24-h wear periods. The demographic characteristics of the participants are summarised in Table 3.

**Table 3:** Participant characteristics

Characteristics	Overall
Age at participation, mean (SD) (years)	62 (12)
Survival length, mean (SD) (years)	3 (3)
Long survivor > 8 years (%)	2 (20)
Males, *n* (%)	8 (80)
Right handedness (%)	9 (90)
MND sub-type, no. (%)*	
ALS	5 (50)
PLS	2 (20)
ND	3 (30)
Bulbar onset (%)	1 (10)
Current intervention use (%)	
Riluzole	1 (10)
Non-invasive ventilation	0 (0)
Gastrostomy	0 (0)
ALSFRS-R at baseline	
Mean	40
Range	31–46
SD	6
Edinburgh Cognitive and Behavioural Screen (ECAS)	
Range	88–125
Mean (SD)	112 (11)

*MND Motor neuron disease, *ALS* amyotrophic lateral sclerosis, *PLS* progressive lateral sclerosis, *PBP* progressive bulbar palsy, *PMA* progressive muscular atrophy, *ND* no data

### Participant expectations of devices

Table 4 reports the responses to Questionnaire 1, exploring participant expectations of the study devices and experiences with technology to monitor their health. All participants reported they were excited about the prospect of trying new technology and the majority (90%) felt that wearable devices were a useful option to track changes in symptoms. 70% had used some sort of wearable device before, and all were supportive that using these devices may mean less appointments in the clinic in the future. No participants reported concerns over the time commitment or extra appointments relating to participation, or concerns regarding side effects or possible interference on their lives of using devices. No participants were concerned about being able to use the devices independently, and there were no concerns that they would be adding burden to their caregivers or would struggle to remember to wear the devices.

**Table 4:** Questionnaire 1: responses of participant expectations of devices

Response item	Total *N* of responses		
	Yes	No	Unsure
I think wearable devices will be a useful option to track changes in my physical symptoms	9	0	1
I have used wearable devices before (e.g. blood pressure checker, step counter, and heart-rate monitor)	7	3	0
I am happy that using devices means I do not have to attend as many trial appointments in the clinic	7	1	2
I am excited about the prospective of trying new technology	10	0	0
I am concerned about the additional time commitment required for participation in this study	0	10	0
I am concerned that participating in this study will mean I have extra appointments to remember	0	10	0
I am concerned about wearing devices and batteries near my skin	0	10	0
I am concerned that wearable devices may interfere with my daily activities	0	10	0
I am concerned that I will not remember to wear the device	0	10	0
I am concerned that I will not be able to work the device as I do not feel confident with new technology	0	10	0
I am concerned that remembering to use the device will be extra work for my caregiver	0	10	0
I am concerned that I may struggle to put on and take off the devices without help	0	10	0
I am concerned about possible side effects from wearing the devices	0	10	0

### Participant feedback on devices

Table 5 summarises participant responses from the questionnaires exploring their experience of study devices. 80% of the participants found wearing the devices to be a positive experience. Comments on the expectations and opinions of the participants towards digital health research and wearable devices are presented in Table 6. 90% of the participants reported they would be happy to wear the devices for a longer time period than the 12 weeks. 60% of the participants indicated that they would be happy to continue wearing the device overnight, which then increased to 80% by the end of the study. By the 12-week time-point, only one person reported disturbed sleep due to wearing the devices overnight.

**Table 5:** Questionnaire 2 and 3: participant experiences of devices

Response item	Total *N* of responses						
	Questionnaire 2: week 6				Questionnaire 3: week 12		
	Yes	No	Unsure	No data	Yes	No	Unsure
I have found wearing the devices to be a positive experience	8	0	2	0	8	0	2
I was happy to wear a device on my wrist	10	0	0	0	10	0	0
I was happy to wear a device on my ankle	10	0	0	0	9	1	0
I would be happy to wear a device for longer time periods (eg several days)	9	0	1	0	9	0	1
I would be happy to wear a device overnight for longer time periods (eg several nights)	6	0	4	0	8	0	2
I found wearable devices to be a helpful option for monitoring my physical MND symptoms	5	1	4	0	3	0	7
I found wearing a device overnight disturbed my sleep	1	9	0	0	1	9	0
I preferred wearing a device to completing regular questionnaires about my physical symptoms	8	1	1	0	5	0	5
I am supportive of the use of wearable devices for tracking physical symptoms	9	1	0	0	10	0	0
I enjoyed the opportunity to try a new device	10	0	0	0	10	0	0
I found wearing a device inconvenient or uncomfortable	1	9	0	0	1	9	0
I needed help to put on and take off the devices	1	9	0	0	1	9	0
I felt that wearing a device has added more responsibilities onto my caregivers	0	9	0	1	0	9	1
I found wearing a device interfered with my daily activities	0	10	0	0	1	9	0
I have found the additional appointments from participating in this study to be inconvenient	0	10	0	0	0	10	0
I have experienced technical issues with my device	1	9	0	0	3	7	0
I struggled to remember to charge the device	0	10	0	0	0	10	0
I have experienced some side effects/problems from wearing the devices	0	10	0	0	0	10	0

**Table 6:** Participants comments on devices

Content theme	Comments to illustrate
Positive comments on using technology in research	Technology is the way ahead
	Happy to carry on
	Encourages movement and keeps me more active
	Wearable devices would be helpful as feeling he is being followed up/monitored and not just left for his MND to progress
	Good for family as well
Feedback on devices and data	He found the device too ‘chunky’ and quite large to wear
	Would like to receive feedback from wearing the devices
Enjoyment of research participation	Happy to help out on any trial for MND
	Enjoyed the experience!
	No problems and happy to be in the study
	Happy to take part if it helps MND research
Difficulty using wrist and ankle straps	People without carers might struggle to use this because buckles are difficult to use. Perhaps Velcro watches would be better
	Practicalities of putting the straps on, there is too much of the strap that is left loose and can catch on things
	Could maybe use an elastic strap/magnetic strap
	Ankle strap is a bit more uncomfortable because of the excess strap, forms a lump on the ankle and is a bit annoying especially overnight
Concerns of device accuracy	Not sure how the devices will assess muscle weakness as opposed to muscle activity
	Unsure of how what comes off of the device will match with his MND symptoms
Impact on sleep	Worse sleep when wearing devices
	Can be slightly uncomfortable at night on the ankle but did not interfere with sleep
Faults with technology	Faulty charger
	Wrist strap broke
	Charger sometimes did not work
	Casing from wrist strap has broken—needs to be attached with tape

No participants reported any side effects, concerns over remembering to charge the devices, interference with daily activities or additional burden from study participation. Despite one individual reporting at both time-points that they found the devices inconvenient or uncomfortable, all participants remained happy that they had the opportunity to try a new device.

At the 6-week time-point, only one individual had experienced technical issues with their device; however, this had risen to 30% of the participants by week 12, an important consideration when considering future studies with potentially larger samples. These technical issues primarily centred around difficulties charging the devices, with the ActiGraph GT9X itself often not connecting to the charging ports in the docking station provided.

### Wear time as an indicator of adherence

Adherence to the protocol of study visits and wear periods was excellent, with participants wearing their wrist devices for a median of 95.7% of the requested time, and 87.3% for the ankle devices, with an overview provided in Table 7. In addition, median wear-time steadily improved over the time period, indicating a high-level of engagement from our participants. A Wilcoxon signed-rank test indicated that wear time in wrist and ankle were significantly different, *T* = 96, *z* = − 5.56, *p* = 2.663e− 08, with greater adherence to the wrist-worn devices. With ten participants using two devices each, across six assessment time-points, 120 periods of 24-h wear were collected. Each of these 120 periods of 24-h wear included 1440 min, providing a total of 172,800 min of data for analysis. Overall, 38% of these assessment periods (*n* = 46) were fully complete with no missing minutes of data. Although wear time is a vendor provided metric and cannot distinguish between periods of wear and non-wear with complete certainty, these initial findings on device use are a promising indication of user engagement.

**Table 7:** Median wear time for 24-h time periods

Device location	Week 0 (min)	Week 12 (min)	Total wear time (min)
Wrist	1338	1440	1378
Range	1162–1440	1229–1440	1080–1440
Ankle	1212	1277	1257
Range	886–1440	1035–1440	755–1440

### Comparing ActiGraph outputs with ALSFRS-R

Variation in ALSFRS-R scores taken at the beginning and end of the study are outlined in Table 8. Daily activity across the 24-h wear periods were measured in the form of VM counts, combining accelerometer sensor data from both wrist and ankle-worn ActiGraph devices, to provide an overall datapoint to represent general physical activity.

**Table 8:** ALSFRS-R scores across the 12-week study period

Study ID	Total score at week 0	Total score at week 12	Difference in scores*
WMND-001	40	44	− 4
WMND-002	46	46	0
WMND-003	31	29	2
WMND-004	44	40	4
WMND-005	45	44	1
WMND-006	29	32	− 3
WMND-007	42	44	− 2
WMND-008	44	41	3
WMND-009	46	46	0
WMND-010	37	30	7

*A negative difference in scores indicates an increased (improved) score

**Fig. 1:**
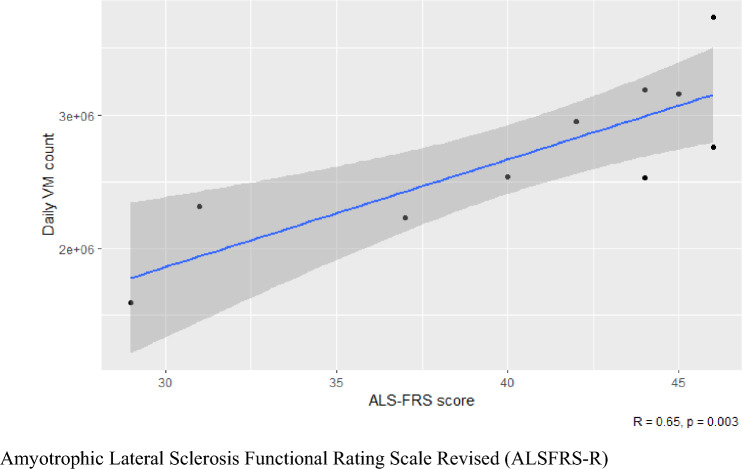
Initial ALSFRS-R score compared with daily activity count at week 0. Amyotrophic lateral sclerosis functional rating scale revised (ALSFRS-R)

A higher VM count denotes more daily physical activity. VM counts from the 24-h wear period at baseline were compared with ALSFRS-R scores at baseline. At baseline, ALSFRS-R correlated with VM count, (*R*^2^ = 0.65, *F*-statistic = 17.93, *p* = 0.003), as shown in Fig. 1. This suggests that those individuals with a higher baseline ALSFRS-R, indicative of better physical function, also had higher VM counts, which is in turn suggestive of being more physically active.

**Fig. 2:**
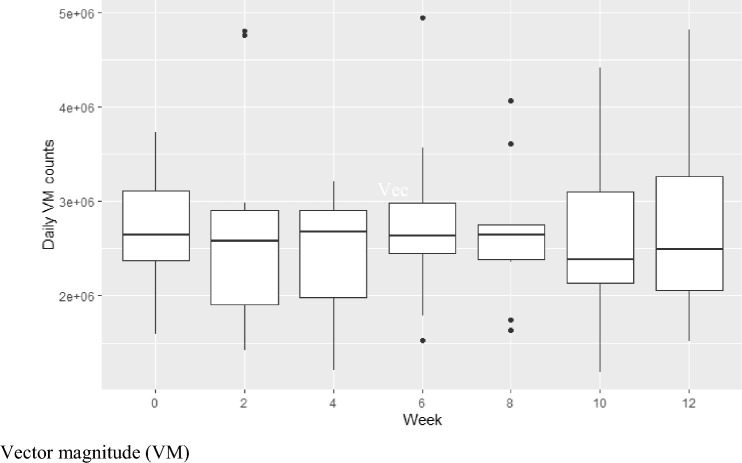
Median daily activity count measured every fortnight for 12 weeks. *VM* Vector magnitude

Participants continued to wear the devices for a total of 6, 24-h wear periods across the 12-week study and level of daily activity at each period is represented by the VM counts in Fig. 2. Overall, participants showed no overall decrease in VM counts over the 12-week period, suggesting that the level of daily activity remained consistent at each of the 6 assessment points.

Mean ALSFRS-R scores for the participants also remained consistent across the study period, from 40.4 at baseline to 39.6 at the end of the study; however, at an individual level, five of the study participants did show decline in their ALSFRS-R score, as highlighted in Table 8. Three participants in fact demonstrated an improvement in their ALSFRS-R scores, highlighting the fallibility of this measure as an endpoint and the inability of this scale to mirror the progressive nature of MND [20].

### ActiGraph association with clinical decline

**Fig. 3:**
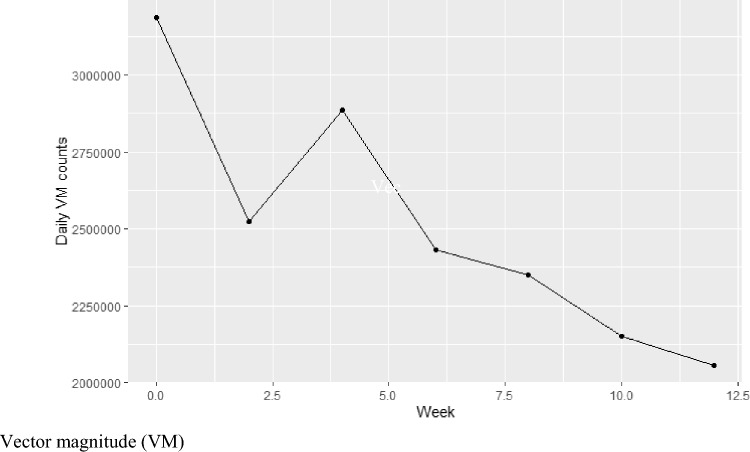
Daily activity count every fortnight for 12 weeks in 1 individual exhibiting notable clinical decline. *VM* vector magnitude

One participant was noted to have a particularly evident clinical deterioration in function, supported by a decline in their ALSFRS-R score from 44 to 40, and a decrease in 6MWT distance from 202 to 166 m during the 12-week study period. Figure 3 shows this participant’s VM counts over the 12-week period, showing a clear decline in physical activity across the 24-h wear periods. This is in contrast with the remaining participants, whose VM counts did not indicate deterioration, raising concerns about the sensitivity of this combination outcome measure to detect decline.

### Six-minute walking test (6MWT)

**Fig. 4:**
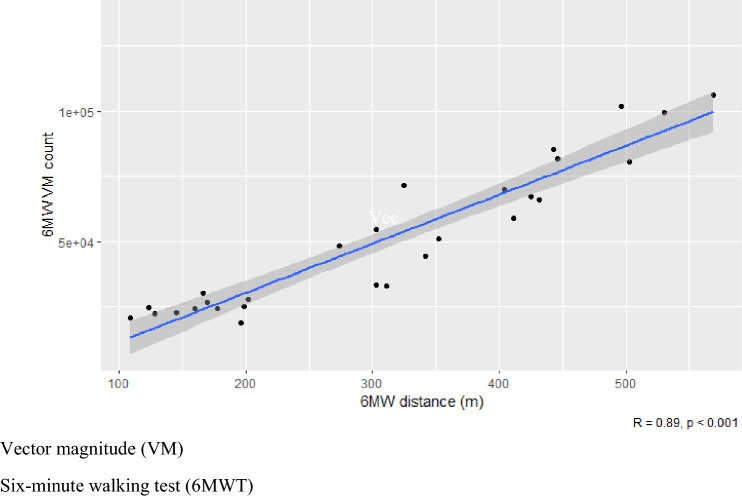
Distance walked in metres during 6MWT compared with VM count on ankle device. *VM* Vector magnitude, *6MWT* 6-min walking test

**Fig. 5:**
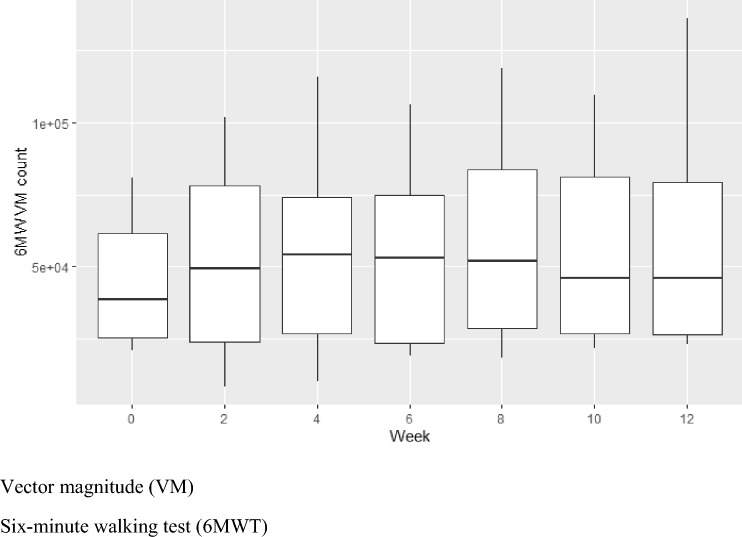
VM count during 6MWT during fortnightly assessments. *VM* vector magnitude, *6MWT* 6-min walking test

This study assessed the acceptability, and suitability, of the ActiGraph devices for use during a validated measure of walking capacity in MND, the 6MWT [11]. During in-person visits, the distance walked was measured, with the distances for each participant across the study shown in Table 9. Total VM counts from the ankle devices, worn during the 6MWT, significantly correlated with distance participants walked in the 6 min when evaluated with repeated measures correlation technique, (*R*^2^ 0.89, *F*-statistic 213.5, *p* < 0.001) (shown in Fig. 4), with higher VM counts associated with a higher distance covered in a 6MWT. VM counts from an accelerometery device, worn during a 6MWT or real-world walking activity, could be a suitable, and sensitive, measure of an individual’s walking capacity and lower limb functionality. As highlighted in Fig. 5, VM counts during the 6MWTs did not decrease over the 12-week study period, indicating no notable decline across the 10 study participants’ distance walked and lower limb functional ability overall.

**Table 9:** Six-minute walk distances

Study ID	Week 0 Distance walked (m)	Week 6 Distance walked (m)	Week 12 Distance walked (m)
WMND-001	311	425	411
WMND-002	503	569	530
WMND-003	178	196	199
WMND-004	202	169	166
WMND-005	432	443	446
WMND-006	109	128	123
WMND-007	342	352	303
WMND-008	274	303	325
WMND-009	404	No data	497
WMND-010	160	145	No data

### In-person and remote accelerometer assessments

**Fig. 6:**
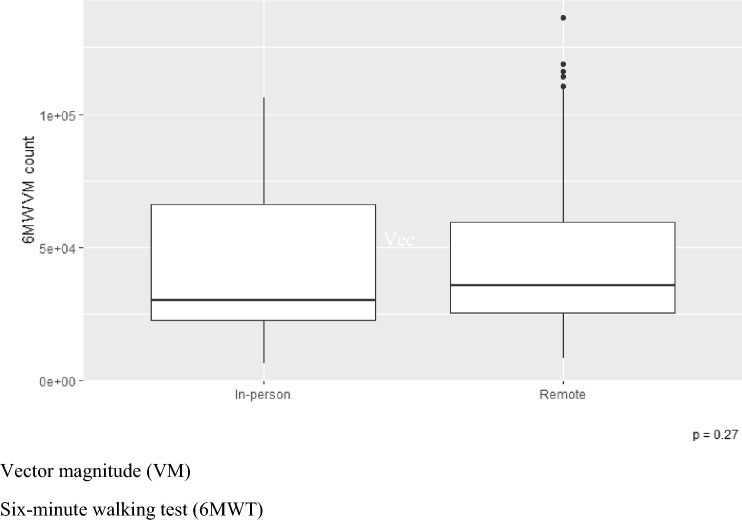
VM count during 6MWT. A comparison of in-person and remote assessment. *VM* Vector magnitude, *6MWT* 6-min walking test

Ankle VM counts were compared between in-person and remote visits where the 6MWT was completed and did not significantly differ (Wilcoxon rank-sum test, *W* = 998, *p* = 0.27) (explored in Fig. 6). This suggests that the 6MWT can be completed remotely, whilst participants are wearing the ActiGraph GT9X devices, and the VM count data remain consistent irrespective of whether it is gathered in an in-person or remote visit.

## Discussion

### Key findings

Ten people with MND wore an ActiGraph GT9X device, with the accelerometer sensor enabled, on their right wrist and right ankle for six periods of 24-h wear. Over the 12-week study period participants attended fortnightly appointments with the research team, both in-person and remotely, to complete motor and walking tasks whilst wearing their devices. Participants were also asked to complete questionnaires on their expectations and experiences at the beginning, middle and end of the study period.

All participants completed the full protocol of study visits and 80% of the participants found wearing these devices to be a positive experience and no one reported experiencing side effects, interference with daily living or added burden from participating in a wearable technology study. One individual reported that using the devices increased burden on their caregiver and one person reported wearing the devices overnight interfered with their sleep. By the end of the study, 30% of the participants had experienced technical issues with their devices.

VM counts from the wearable devices correlated with established measures of physical function, the ALSFRS-R and 6MWT distance. There was no overall decline in levels of daily activity over the 12-week study period detected by the ActiGraph GT9X devices’ accelerometers and 50% of participants showed either no decline or an improvement of physical function on established measures of disease progression.

This study provides positive and promising initial feedback on the acceptability of wearable devices in a small group people with MND. In particular, this style of device with wrist and ankle wear locations seems to be initially acceptable for wearing during remote assessments and overnight to evaluate sleep. Data from the accelerometer sensor indicate initial correlations with established measures of function and warrants further investigation.

### Acceptability of wearable devices

The findings from this study suggest that this small sample of people with MND were motivated to engage with research into technology to evaluate health, and generally found wrist and ankle-worn smart-watch style devices acceptable to use during their daily activities and for wear overnight.

The individuals who participated in this study were highly engaged with the potential of digital technology to evaluate health and 70% reported having used wearable devices previously. This is analogous with the findings from a recent study that indicated 82% of their surveyed participants with MND used some form of digital device daily [21].

Our findings add a participant-focussed narrative to the current landscape of digital technology to evaluate motor function in MND, addressing a gap identified in this recent review [22]. Our review found that whilst initial results of the 20 included studies that explored device efficacy were promising, only 36% of studies reported on the acceptability of devices to participants [22].

### Sample bias

When interpreting the findings from this small-sample exploratory study, it is important to remember that these participants represent a sub-set of those affected by MND, and MND is a highly heterogeneous condition, presenting and progressing differently [23]. The ten people who provided feedback on the devices used in this study were motivated to engage with research generally, physically well enough to complete motor tasks, able to travel to clinic and technologically literate enough to attend videoconferencing appointments. When attempting to extrapolate these findings on acceptability, the relatively minimal disease burden and high engagement with research of this sample of people with MND must be considered.

Although the majority of study visits were conducted remotely, participants did attend appointments in-person at the Anne Rowling Regenerative Neurology Clinic in Edinburgh. The requirement to attend a clinical appointment may have been a barrier to participation for some people with MND; however, fully remote delivery brings its own unique challenges in technology studies [24].

Despite being physically capable of attending in-person visits, 70% of the participants identified that the potential for reducing the number of in-person visits was a key benefit of using wearable devices for research. This study provides initially promising data to suggest that wearing devices whilst completing motor tasks and walking tests is an acceptable alternative to in-person research visits for people with MND.

### Feasibility

VM counts (raw accelerometery counts from the 3 axes) from the accelerometer sensors used in this study were found to correlate with the ALSFRS-R; however, the ALSFRS-R’s own issues discussed earlier means that convergent validity with this established measure will be insufficient to ascertain the validity of prospective devices [25]. VM counts from ankle-worn accelerometers correlated with another established measure of MND functional capacity, the 6MWT [18] distances, providing further evidence of these sensor’s outputs convergent validity with established measures.

There was no clear decrease in total VM counts during the 24-h wear periods or in ankle VM counts throughout 6MWT across the 12-week study. This lack of evident decline was also reflected in ALSFRS-R scores, with only one individual declining in the study timeframe on this measure. This lack of evident decline may be due to the study timeframe being too short to detect change, as a shorter study length was chosen to explore the primary aim of acceptance in these study participants.

Although previously accelerometery outputs appear to have some sensitivity to detect changes in functional ability that traditional questionnaire-based assessments may not capture [12], a longer study duration than that used in the current study will be required to provide sufficient data to explore how functional decline can be measured using accelerometers.

### Evaluating sleep

Exploring the acceptability of wearing the ActiGraph GT9X devices during sleep was a key novel aspect of this study. Evaluating people with MND whilst they are sleeping can provide key insights into breathing and physical functioning. Understanding of breathing during sleep offers an opportunity for early intervention with life-improving clinical equipment, for example, providing non-invasive ventilation to reduce the number of times someone is awoken by their respiratory insufficiency. Disturbed or non-refreshing sleep, either due to breathing or movement difficulties, occurring secondary to motor degeneration, can have a significant impact on fatigue, quality of life, prognosis and wellbeing [26].

Wearable devices offer a unique opportunity to evaluate changes in sleep, taking continual or repeated measures of sleep parameters such as time asleep, time to fall asleep, awakenings and movement whilst the wearer is asleep [27]. However, to avoid further disruption to rest, assessing the acceptability of these devices during sleep, distinctly from daytime wear, is important.

By the end of this 12-week study, involving seven nights of data collection, only one participant reported disturbed sleep from wearing these devices. Finding devices which cause minimal interference, whilst still able to collect clinically relevant data, are a delicate balance where feedback from people with MND on acceptability is as crucial as evidence on device efficacy [28].

### Managing technical issues

Concerns over charging the devices were the primary technical problem experienced by participants. The majority of problems reported by participants focussed on practical rather than technical issues; difficulty putting on the devices and faulty straps, with the large size of the device affecting their comfort. The research team experienced several technical issues with the devices. Difficulties with charging devices, connecting devices to computers and problems saving data resulted in frequent concerns that the devices were either not collecting data or that the data were not being accurately transferred to the research team.

A key benefit of conducting small-scale feasibility studies is that these enable us to investigate, identify and addresses such technical and practical issues identified by participants and researchers, before considering implementing any devices in larger studies which require more resources. In larger scale studies with longer timeframes, widespread technical issues may result in attrition, missing data and added burden to participants’ daily lives.

### New directions in ActiGraph

ActiGraph are in the process of developing their next generation of wearable devices, the wrist-worn LEAP. In addition to the three-axis accelerometer, gyroscope and magnometer sensors available in the GT9X, the devices used in the current study, the ActiGraph LEAP will include additional sensors to evaluate vital signs (specifically heart rate, skin temperature and oxygen saturation) and an in-built microphone. The design has also been updated to align with commercially available wrist-worn technology to monitor health, in an attempt to address previous concerns from users over the devices’ larger size and improve the comfort of users.

Unlike the GT9X, the LEAP can remain in the watchstrap during charging, with the weight of the device creating a downward force to ensure the device and charging port remain connected. This will address two key concerns raised by participants in the current study; the fine motor skills required to repeatedly remove the device from the strap, and issues with the device and charging port connecting affecting the ability to charge their device. Increased battery life and improved facility for remote data uploads with the LEAP addresses two key concerns of the research team, the potential for missing data due to charging issues or a requirement for a hard upload of device data.

Due to the change in design, to use these devices new chargers, adapters and watchstraps are required for each participant, raising concerns over the environmental impact [29] and the repeated financial outlay of implementing these new generations. This highlights a broader issue with health technology research; as technology continues to rapidly advance, devices purchased and implemented quickly become obsolete. To provide large cohorts with up-to-date devices is a costly, Sisyphean task.

### Limitations

The primary limitation of this study is the small sample size of ten individuals, and the impact that this may have on the generalisability of the findings. In a condition such as MND that is highly heterogenous in presentation and progression, a smaller sample size may be unable to capture the variability across the disease spectrum and the applicability of any findings to others with the same condition is limited in turn.

Participants in this study may also not be representative of the wider MND population due to their previous experience of using health monitoring technologies, willingness to engage with technology and relatively high technological literacy. These participants were also more broadly interested in contributing to research, and had sufficient health and the social support to attend in-person clinical appointments as a research participant, criteria that may not be applicable to many affected by MND, particularly as their disease progresses.

The smaller sample size, and bias towards more technologically literate people with MND, limits the inferences that we can make from their experiences to the wider population with this condition. However, this study was exploratory in nature and a smaller sample size enabled us to collate initial feedback on the suitability and acceptability of limb-worn wearable devices to group of Scottish people with MND. Through this pilot study, we identified potential areas of concern that would need to be addressed before considering future larger studies requiring more participants and resources.

An additional limitation of this study, and a broader issue in the evaluation process of technologies, is establishing content validity. Understanding what constitutes ‘content’ in motor activity, impairment and decline in people with MND is a subjective form of measurement in itself. Outcomes from the sensors in the study devices may be insufficient to quantify the motor dysfunction experienced. Some measures, particularly when used in isolation, have been shown to be insufficiently sensitive to represent the change in movement strength needed to detect smaller changes in function [30] particularly during periods of activity [31]. Data on the technical validity of the ActiGraph GT9X outcomes in MND are limited [32] and further evidence of content validity is needed to contribute towards the evaluation process of technological assessment tools.

### Future work

A key area that future research can expand upon, alongside responsiveness and acceptability of candidate devices, is the feasibility of accelerometer sensors to measure motor decline. The World Health Organisation’s definition of feasibility includes establishing if a prospective measure works as intended in a given context, evaluating if the devices were able to capture the information required to map onto the motor symptoms as they present and progress. Focussing on the quantity and quality of data collected will enable future researchers to address is accelerometers work as intended in the context of MND research.

The next step towards clinical validation of devices as measures of disease progression, that can be addressed through future research, is concurrent validity with suitable clinical reference standards [12]. In MND the ALSFRS-R is the most widely used clinical reference standard and this measure provides a total score to represent overall motor dysfunction that is generated by summing domain specific sub-scores. Relying only on covariance with total scores to validate prospective devices will limit advancement in this area. Future research needs to also focus on covariance with the sub-domain sub-scores of the ALSFRS-R as this may be more may be more informative than using total summed scores [3].

As discussed in the introduction, although the ALSFRS-R remains to primary outcome measure of many trials it has limitations as a measure of progression [3, 5, 20]. To overcome these flaws, we recommend the comparison of prospective technologies with a number of surrogate measures. This enables future researchers to provide corroborating validation checks and avoid the limitations of any single measure, such as the ALSFRS-R when evaluating digital measures of disease progression in MND. A potential example of this is the 6-min walking test (6MWT) completed by participants in this study. As an established measure of physical function in MND [33], the distance walked during the 6MWT can be used as a surrogate outcome measure, alongside other measures of function, to evaluate covariance with data obtained from prospective technologies.

Further research into the optimal wear locations, both for accelerometer sensors and devices evaluating other aspects of health, is needed to inform recommendations. A more detailed exploration of how different wear locations are comparable in the data provided is needed, particularly in MND, as the progression of MND is heterogenous. Devices placed on different locations on the body will likely be measuring different constructs of physical decline, and we recommend analysing the data separately if multiple wear locations are used. The acceptability of these different wear locations to participants with MND must also be considered when developing recommendations. A comparison of the potential burden experienced when using multiple devices concurrently in different locations, or preferences for specific wear locations, must be included when considering recommending any wear locations. The acceptability of candidate devices in different wear locations will be a key aspect of future research, to improve our understand of how the participant’s acceptance of devices differs depending on where they are asked to wear them.

In the current study, participants wore a device on their wrist and ankle; however, future research may explore the potential of different wear locations that new developments in health technology offer. Rings, ear-bud headphones, headbands, glasses and smart clothing are just some of the continuous innovations in technology that may help us to better understand and support the needs of people with MND. Regardless of the device, feedback on the acceptability of all aspects of prospective devices must be prioritised when evaluating the suitability for people with MND. This can be done alongside, or prior to, exploring a devices’ efficacy in diagnosing and monitoring MND. Including feedback from prospective users is a key element to making informed decisions on device selection and it may be beneficial to repeat these evaluations at multiple time-points to understand how wear experience differs and changes.

## Conclusion

Validation of a prospective technology is an iterative process involving multiple stages of evaluation and assessment, with no universal conclusive outcome as device specifications and participant needs change. The feedback our participants provided on their experience of using a wrist and ankle-worn devices during remote study visits, whilst undertaking their usual daily activities and overnight whilst asleep, will add a participant-focussed perspective to this evaluation process. We recommend that any future studies contributing to the validation of digital health technologies include an assessment of the acceptability of the technologies to prospective users. The comparability of these findings on acceptance across different studies can be improved through the use of validated questionnaires [34].

Our findings indicate initially promising acceptance of wearable devices to evaluate motor symptoms in people with MND. However, these inferences must be made with the caveat that the small number of participants in this exploratory study were a sub-set of individuals with MND who were physically and logistically able to engage in this study, and also expressed a particular interest in using technology for health monitoring. Many people with MND would not fit this description, and future research design must strive to be inclusive of those who may be at risk of digital exclusion due to their health, social support network or technological literacy.

The frequency of technical issues experienced with the ActiGraph GT9X was a key concern with this specific device. Whilst including technology-based outcome measures offers the opportunity to address many of the barriers previously affecting research engagement, it also introduces new concerns regarding equity of access. The next generation of the ActiGraph devices, the LEAP, offers a new design that will hopefully address many of the technological issues experienced in our study.

The findings from this small study contribute promising feedback from people with MND about the potential suitability of these types of devices, but we must explore this further by continuing to prioritise participant-experience alongside any future work evaluating device efficacy.

## Data Availability

Data are available as a supplementary file to this manuscript, with identifying information removed.

## Appendix 1: Questionnaires

### Questionnaire 1: participant expectations at screening (0 week time-point)

**Table:**

•Thank you for your involvement in this study on movement devices, your contribution to research is very much appreciated				
• Please complete the following short questionnaire about your experience with using wearable devices for monitoring symptoms				
• Please listen to the statements and indicate if they are important to you				
• There is a comment box at the end to add any other factors				
• If there are any statements you do not wish to comment on, please choose the 'Prefer Not to Say' option				
	Yes	No	Unsure	Prefer not to say
I think wearable devices will be a useful option to track changes in my physical symptoms				
I have used wearable devices before (e.g. blood pressure checker, step counter, heart-rate monitor)				
I am happy that using devices means I do not have to attend as many trial appointments in the clinic				
I am excited about the prospective of trying new technology				
I am concerned about the additional time commitment required for participation in this study				
I am concerned that participating in this study will mean I have extra appointments to remember				
I am concerned about wearing devices and batteries near my skin				
I am concerned that wearable devices may interfere with my daily activities				
I am concerned that I will not remember to wear the device				
I am concerned that I will not be able to work the device as I do not feel confident with new technology				
I am concerned that remembering to use the device will be extra work for my caregiver				
I am concerned that I may struggle to put on and take off the devices without help				
I am concerned about possible side effects from wearing the devices				
Do you have any other expectations or thoughts about wearing a device?				

### Questionnaire 2: participant evaluations mid study (6 week time-point)

**Table:**

• Thank you for your involvement in this study on movement devices, your contribution to research is very much appreciated				
• Please complete the following short questionnaire about your experience with using wearable devices for monitoring symptoms				
• Please listen to the statements and indicate if they are important to you				
• There is a comment box at the end to add any other factors				
• If there are any statements you do not wish to comment on, please choose the 'Prefer Not to Say' option				
	Yes	No	Unsure	Prefer not to say
I have found wearing the devices to be a positive experience				
I was happy to wear a device on my wrist				
I was happy to wear a device on my ankle				
I would be happy to wear a device for longer time periods (eg several days)				
I would be happy to wear a device overnight for longer time periods (eg several nights)				
I found wearable devices to be a helpful option for monitoring my physical MND symptoms				
I found wearing a device overnight disturbed my sleep				
I preferred wearing a device to completing regular questionnaires about my physical symptoms				
I am supportive of the use of wearable devices for tracking physical symptoms				
I enjoyed the opportunity to try a new device				
I found wearing a device inconvenient or uncomfortable				
I needed help to put on and take off the devices				
I felt that wearing a device has added more responsibilities onto my caregivers				
I found wearing a device interfered with my daily activities				
I have found the additional appointments from participating in this study to be inconvenient				
I have experienced technical issues with my device				
I struggled to remember to charge the device				
I have experienced some side-effects/problems from wearing the devices				
If you are thinking of leaving the study, is there anything we can help with?				
Did you experience any negative effects of wearing the devices? If so, please list below				
Do you have any other comments about your experience so far of wearing devices?				

### Questionnaire 3: participant evaluations at end of study (12 week time-point)

**Table:**

• Thank you for your involvement in this study on movement devices, your contribution to research is very much appreciated				
• Please complete the following short questionnaire about your experience with using wearable devices for monitoring symptoms				
• Please listen to the statements and indicate if they are important to you				
• There is a comment box at the end to add any other factors				
• If there are any statements you do not wish to comment on, please choose the 'Prefer Not to Say' option				
	Yes	No	Unsure	Prefer not to say
I have found wearing a device to be a positive experience				
I was happy to wear a device on my wrist				
I was happy to wear a device on my ankle				
I would be happy to wear a device for longer time periods (eg several days)				
I would be happy to wear a device overnight for a longer time period (eg several nights)				
I found wearable devices to be a helpful option for monitoring my physical MND symptoms				
I preferred wearing a device to completing regular questionnaires about my physical symptoms				
I am supportive of the use of wearable devices for tracking physical symptoms				
I found wearing a device overnight disturbed my sleep				
I enjoyed the opportunity to try a new device				
I found wearing a device inconvenient or uncomfortable				
I needed help to put on and take off the devices				
I felt that wearing a device has added more responsibilities onto my caregivers				
I found wearing a device interfered with my daily activities				
I have found the additional appointments from participating in this study to be inconvenient				
I have experienced technical issues with my device				
I struggled to remember to charge the device				
I have experienced some side-effects/problems from wearing the devices				
Did you experience any negative effects of wearing the devices? If so, please list below				
Do you have any other comments about your experience of wearing devices?				

## Appendix 2: ALSFRS-R

### Speech

**Table:**

How is your speech?		
Please tick		
1	My speech is normal, there has been no change since my diagnosis	
2	There is a change in my speech that people (either yourself or others) have noticed	
3	People can understand me when I speak but I have to repeat myself often (around a quarter of the time, or more)	
4	I can speak but I also need to use technology or writing to be understood	
5	I can no longer speak	

### Saliva

**Table:**

How is your saliva?		
Please tick		
1	I do not have any excessive saliva (please still tick this option if you have a dry mouth	
2	I feel I have excessive saliva and there may be some drooling at night, but I do not usually have to mop up saliva with a tissue	
3	I need a use a tissue to mop up excessive saliva, but not often (less than a quarter of the time)	
4	I experience drooling and have to use a tissue to mop up excessive saliva often, but not all the time	
5	I need to use a tissue, or suction device, to mop up excessive saliva all of the time	

### Swallowing

**Table:**

How is your swallowing?		
Please tick		
1	I have no problem eating food and having drinks the same as I did before my MND diagnosis	
2	I have some issues with food sticking in my throat or coughing/choking when I eat. Sometimes I have to cut food up small, but I never have to mash or liquidise food	
3	I need to have food mashed or liquidised, or my drinks need thickeners in. I avoid tougher and drier foods	
4	I struggle to eat food and I need to have gastrostomy to add to my calories intake (please tick yes if you need gastrostomy, regardless of if you have one or not)	
5	I only take in calories through gastrostomy, or other supported feeding	

### Handwriting

**Table:**

Thinking about your dominant hand, are you able to hold a pen? Please tell us about your handwriting		
1	My handwriting is normal there has been no change	
2	My handwriting is slower and sloppier but all words are legible, or sometimes I use writing aids or specialised pen grips	
3	Not all of my words are legible when I write	
4	I can grip a pen, but my words are not legible	
5	I am unable to grip a pen	

### A. Cutting food and handling utensils—without gastrostomy

**Table:**

Please complete this section is you do not use gastrostomy as your only source of calories. If you only use gastrostomy, please move on to question 5B below. How are you with cutting food or handling cutlery?		
1	There has been no change in my ability to cut up my food and I have not changed the utensils I use to eat	
2	I am a little slow and clumsy, but I do not need help from others	
3	Occasionally I need help from others to eat, but usually I can do it alone	
4	I need someone else to cut up my food, but I am able to eat independently	
5	I need some to cut up my food and help me with eating	

### B. Cutting food and handling utensils—with gastrostomy

**Table:**

If you do not have gastrostomy, or still have some food, please complete 5A instead. How are you with handling the gastrostomy fastenings and fixtures?		
1	I have no difficulty with setting up the fastenings and fixtures	
2	I am a little slower and clumsy but I can do it myself	
3	I need some help from others with the closures and fasteners	
4	I need someone else to do most of the setting up	
5	I am unable to do the task alone, someone else has to do it for me	

### Dressing and hygiene

**Table:**

How are you with dressing or washing?		
1	There has been no change since my MND began	
2	I am a little slower but I do not need other people or supportive devices (eg button hook) to help me	
3	I need some assistance at some stages from other people, or supportive devices, when I am getting dressed or washing, but I can do most of it myself	
4	I need assistance when I am getting dressed or washing, but I can do some parts myself	
5	I cannot dress or bathe myself and need support from others	

### Turning in bed and adjusting bed clothes

**Table:**

Can you turn in bed and adjust the bed clothes? (pillow/blankets)		
1	I can do this as normal	
2	I am a little slower and clumsy but I can do this without help	
3	I can turn, or adjust the bed sheets, alone but with great difficulty	
4	I can begin to turn or adjust the bed sheets but I need someone to help me	
5	I cannot turn or adjust the bedsheets	

### Walking

**Table:**

How is your walking?		
1	I can walk as normal	
2	I am have some issues with walking slowly, tripping or balance but I do not need help from others or a walking aid	
3	I can walk with help from others or a walking aid	
4	I can stand up and weight bear	
5	I can no longer walk	

### Climbing up stairs

**Table:**

Are you able to climb up stairs? Please only think about going up, not down		
1	I have no problems going up stairs	
2	I am a little slower but I do not feel unsteady or need to rest between steps	
3	I need to rest between steps and I feel unsteady	
4	I need support from another person or a handrail to be able to climb stairs	
5	I cannot climb stairs	

### Breathlessness

**Table:**

Do you become breathless? If you are using non-invasive ventilation please choose the final option		
1	No I do not become breathless	
2	I get breathless when I am walking (walking at a good speed on a flat route)	
3	I get breathless when I am eating, bathing or dressing	
4	I get breathless when I am sitting or lying down	
5	I have significant difficulty breathing and I either currently use, or am considering using, a ventilation system	

### Lying down

**Table:**

Can you sleep lying down flat or do you need to be propped up?		
1	I do not need to be propped up	
2	I have some difficulty sleeping at night due to shortness of breath, but I do not routinely use more than two pillows	
3	I need more than two pillows to sleep, or I need to be angled at 45 degrees or more	
4	I can only sleep sitting up in bed or a chair	
5	I can only sleep with non-invasive mechanical ventilation on for most, or all, of the night	

### Respiratory insufficiency

**Table:**

Do you use non-invasive ventilation?		
1	I do not use	
2	I use ventilation occasionally	
3	I use ventilation during the night only	
4	I use ventilation during the day and night	
5	I have permanent invasive ventilation by intubation or tracheostomy	

## Appendix 3: CARE-MND clinical data requested

In addition to data collection through questionnaires, this project involved a data request to CARE-MND for clinical information on participants.

**Table:**

Area	Data
Demographics	Age at diagnosis
	Sex
	Date of diagnosis
	Date of death
Phenotype	MND subtype
	Site of onset
	Areas currently affected
Interventions use	Riluzole
	Non-invasive ventilation
	Gastrointestinal feeding tube insertion
Speech	Referral to Speech and Language Therapist
	Sialorrhea
	Problematic saliva
Physical symptoms	Additional ALSFRS-R data where required
	Area of symptom onset
